# Orf virus infection in Alaskan mountain goats, Dall’s sheep, muskoxen, caribou and Sitka black-tailed deer

**DOI:** 10.1186/s13028-018-0366-8

**Published:** 2018-02-21

**Authors:** Morten Tryland, Kimberlee Beth Beckmen, Kathleen Ann Burek-Huntington, Eva Marie Breines, Joern Klein

**Affiliations:** 1 Department of Arctic and Marine Biology, Arctic Infection Biology, UiT-Arctic University of Norway, Framstredet 39, 9019 Tromsø, Norway; 2 Alaska Department of Fish and Game, Division of Wildlife Conservation, 1300 College Road, Fairbanks, AK 99701 USA; 3 Alaska Veterinary Pathology Services, 23834 The Clearing Drive, Eagle River, AK 99577 USA; 4 Department of Nursing and Health Sciences-Vestfold, Faculty of Health and Social Sciences, University College of Southeast Norway, 3603 Kongsberg, Norway

**Keywords:** Alaska, Caribou, Contagious ecthyma, Dall’s sheep, Deer, Mountain goat, Muskox, Parapoxvirus, Virology, Wildlife, Zoonosis

## Abstract

**Background:**

The zoonotic Orf virus (ORFV; genus *Parapoxvirus*, *Poxviridae* family) occurs worldwide and is transmitted between sheep and goats, wildlife and man. Archived tissue samples from 16 Alaskan wildlife cases, representing mountain goat (*Oreamnos americanus*, n = 8), Dall’s sheep (*Ovis dalli dalli*, n = 3), muskox (*Ovibos moschatus*, n = 3), Sitka black-tailed deer (*Odocoileus hemionus sitkensis*, n = 1) and caribou (*Rangifer tarandus granti*, n = 1), were analyzed.

**Results:**

Clinical signs and pathology were most severe in mountain goats, affecting most mucocutaneous regions, including palpebrae, nares, lips, anus, prepuce or vulva, as well as coronary bands. The proliferative masses were solid and nodular, covered by dark friable crusts. For Dall’s sheep lambs and juveniles, the gross lesions were similar to those of mountain goats, but not as extensive. The muskoxen displayed ulcerative lesions on the legs. The caribou had two ulcerative lesions on the upper lip, as well as lesions on the distal part of the legs, around the main and dew claws. A large hairless spherical mass, with the characteristics of a fibroma, was sampled from a Sitka black-tailed deer, which did not show proliferative lesions typical of an ORFV infection. Polymerase chain reaction analyses for *B2L*, *GIF*, *vIL*-*10* and *ATI* demonstrated ORFV specific DNA in all cases. Sequences from Dall’s sheep formed a separate cluster, comparable to ORFV from domestic sheep. Sequences from the other species were different from the Dall’s sheep sequences, but almost identical to each other.

**Conclusions:**

This is the first major investigation of parapoxvirus infections in large Alaskan game species, and the first report of parapoxvirus infection in caribou and Sitka black-tailed deer. This study shows that most of the wild ruminant species in Alaska and from most parts of Alaska, can carry and be affected by ORFV. These findings call for attention to transmission of ORFV from wildlife to livestock and to hunters, subsistence harvesters, and wildlife biologists.

**Electronic supplementary material:**

The online version of this article (10.1186/s13028-018-0366-8) contains supplementary material, which is available to authorized users.

## Background

Orf virus (ORFV) is the type species of the genus *Parapoxvirus* of the *Poxviridae* family, which also includes Bovine papular stomatitis virus, Pseudocowpox virus and Parapoxvirus of red deer in New Zealand [1]. ORFV is distributed worldwide with domestic sheep and goats, causing contagious ecthyma (CE) (*syn*. contagious pustular dermatitis, scabby mouth). ORFV produce lesions in the skin and at muco-cutaneous interface at the muzzle, lips, eyelids, anus, prepuce or vulva, and teats as well as in the oral mucosa, such as the tongue and the gingiva, especially associated with erupting teeth [2]. CE typically starts with a macule at the port of entry, progressing to a papule and pustule. Since ORFV express the proteins homologous to endothelial growth factor and vascular permeability factor, both being angiogenesis regulators [3], such lesions becomes highly vascularized and proliferative (“cauliflower-like”) and are easily exposed to trauma and bleeding, followed by secondary bacterial infections. Chronic CE lesions are thus often presented as masses covered by thick crusts (skin) or as proliferative masses (mucosa). Histological changes are present in the epithelium as ballooning degeneration, reticular degeneration, marked epidermal proliferation and epidermal micro-abscesses [4].

CE, often referred to as “contagious ecthyma-like” when lacking a virological confirmation, has been reported in many free ranging animal species [5], such as semi-domesticated reindeer (*Rangifer tarandus tarandus*) [6–8], captive and wild muskoxen (*Ovibos moschatus*) [9–11], and red deer (*Cervus elaphus*) [12, 13].

In Alaska, CE has been described in the domestic sheep and goats, as well as in wild Dall’s sheep (*Ovis dalli dalli*), mountain goat (*Oreamnos americanus*) and muskox [9, 14]. Severe outbreaks have also been reported in Alaskan captive muskox [15], and it has been shown experimentally that also moose (*Alces alces*) and caribou (*Rangifer tarandus*) calves are susceptible to infection with ORFV isolated from sheep [9].

Since ORFV is zoonotic, these infections represent an occupational hazard for people handling affected animals, such as veterinarians, sheep herders, hunters and game managers [16–18]. In the scientific literature, one human case has been reported from Alaska, with ORFV transmitted from a mountain goat [19]. ORFV is probably one of the most common poxvirus infection in humans, causing lesions mainly on fingers and hands. In most patients, a single papule appears which may develop into a large vesicle or bulla, sometimes being hemorrhagic, with a black appearance. The condition can be associated with by local lymphadenopathy and mild fever and is usually mildly painful, but ORFV infections may also become generalized and severe, especially in immunocompromised persons [20]. Humans are infected through direct contact with infected animals, including wildlife [16, 19]. ORFV infection is well known among farmers and the disease is listed as one that people of the Arctic should be aware of through contact with infected game species [21].

A large number of game is harvested in Alaska each year, by indigenous people as part of their tradition and culture, and by local and non-resident hunters. In 2015, 15,554 Sitka black-tailed deer, 4332 caribou, 256 muskoxen, 528 mountain goats, and 11 Dall’s sheep were reported harvested in Alaska [22], indicating that people, to a substantial degree, are in close contact with these large game species.

The aims of this study were to verify the preliminary CE diagnoses in 16 wildlife cases by identifying the causative virus. We also wanted to characterize this zoonotic disease in wild ruminant game species in Alaska, and conduct a phylogenetic comparison of the circulating viruses with known reference viruses of the *Parapoxvirus* genus.

## Methods

### Clinical cases and sampling

**Fig. 1:**
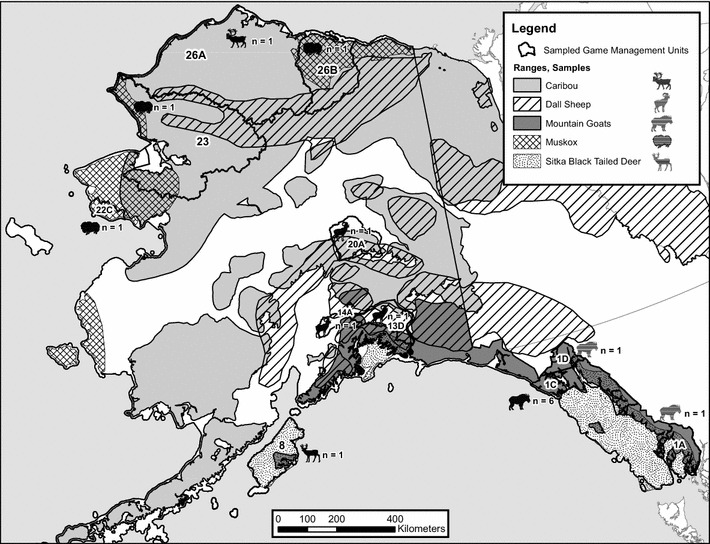
Map of Alaska showing the geographical distribution of the animal species and the location and numbers of the examined cases of contagious ecthyma in mountain goat (*Oreamnos americanus*), Dall’s sheep (*Ovis dalli dalli*), muskox (*Ovibos moschatus*), caribou (*Rangifer tarandus granti*) and Sitka black-tailed deer (*Odocoileus hemionus sitkensis*) (see Table 1 for identification of each case to game management unit; GMU)

Fifteen animals (Table 1) that during the period 2002–2012 were hunted, reported dead or euthanized for animal welfare reasons were subjected to our investigations. They had lesions in the mucocutaneous interface and/or on coronary bands consistent with or suspicious of CE. One additional animal (Sitka black-tailed deer) had lesions consistent with a fibroma, although CE was mentioned as a differential diagnosis in the necropsy report. The geographical location of the clinical cases is depicted in Fig. 1. The animals were subjected to field dressing, and whole carcasses or body parts were sent to Alaska Department of Fish and Game, Fairbanks, Alaska, for diagnostic investigations. Tissue samples from gross lesions were obtained during necropsies, using aseptic techniques and stored in Whirl Pac (Nasco, Medesto CA, USA) or cryogenic vials at − 20 °C or colder. A subset of these frozen tissue samples (20 mg each) was obtained from three different areas from each case, from areas showing gross pathological changes consistent with CE, using aseptic techniques to avoid contamination between samples. These subsamples were subjected to further virological investigations. Additional tissue sections were preserved in 10% neutral buffered formalin.

**Table 1:** Alaskan ruminants (case 1–16) with contagious ecthyma: accession numbers in GenBank, biological data, geographical origin and type of sample investigated for parapoxvirus (see text for pathological findings and Fig. 1 for geographical origin)

Case	Accession no. GenBank^a^	Animal species	Animal ID	Sex	Age	Date of death	Origin (GMU^b^)	Skin/mucosa investigated
1	KJ944487 KM057386 KM098076 KY702590	Mountain goat	2009-008	M	Adult	25 Feb 2009	Mount Juneau (1C)	Nostril, lower lip
2	KJ944499 KM057383 KM098072 KY702586	Mountain goat	2006-029	F	10 months	18 March 2006	Mount Juneau (1C)	Lips, eyelids, ear
3	KJ944496 KM057391 KM098081 KY702595	Mountain goat	2011-018	F	4 year	6 Feb 2011	Haines (1D)	Eyelid, lip, nares
4	KJ944491 KM057382 KM098071 KY702585	Mountain goat	2005-167	F	1 year	2 Oct 2005	Sun Dial Lake Ketchikan (1A)	Lip, nares
5	KJ944497 KM057381 KM098070 KY702584	Mountain goat	2005-064	M	Subadult	1 Dec 2001	Juneau (1C)	Eye lid, neck
6	KJ944494 KM057384 KM098073 KY702587	Mountain goat	2006-217	M	6 months	28 Nov 2006	Mount Juneau (1C)	Nares, lips, ear
7	KJ944501 KM057380 KM098069 KY702589	Mountain goat	2008-136	–	–	–	Not specified (1C)	Not specified
8	KJ44495 KM057385 KM098075 KY702583	Mountain goat	1-29-02	–	–	–	Not specified (1C)	Not specified
9	KJ944490 KM057379 KM098074 KY702588	Dall’s sheep	2007-156	M	2 months	27 Jul 2007	Black Mountain (13D)	Lesion on the lip
10	KJ944489 KM057393 KM098083 KY702597	Dall’s sheep	2011-106	M	2 months	28 Jul 2011	Carpenter Creek (14A)	Leg, face, chin
11	KJ944488 KM057387 KM098077 KY702591	Dall’s sheep	2010-009	NA	Lamb	March 2010	Dry Creek (20A)	Hooves/coronary band
12	KJ944500 KM057388 KM098078 KY702592	Muskox	2010-049	M	5 year	24 April 2010	Sagwon River (26B)	Nares
13	KJ944493 KM057389 KM098079 KY702593	Muskox	2010-147	F	Adult	18 Oct 2010	Kivalina (23)	Nares, interdigital space
14	KJ944498 KM057392 KM098082 KY702596	Muskox	2011-041	F	Adult	4 Oct 2011	Safety Sound Nome (22C)	Hooves/coronary band
15	KJ944492 KM057390 KM098080 KY702594	Sitka black-tailed deer	2011-005	F	Adult	12 Sep 2010	Uyak Bay, Kodiak (8)	Perineum
16	KT821472 KM057394 KM098084 KY702598	Caribou	2012-137	F	14 months	6 Aug 2012	Admiralty Bay/Barrow (26A)	Front legs, upper lip

^a^Given for the genes *B2L*, GM-CFS IL 2 gene (*GIF*), the putative interleukin 10 gene (*vIL10*), and A type inclusion (*ATI*), respectively

^b^GMU: Game Management Unit, Alaska

### Histopathology

Formalin fixed tissues were processed routinely, and paraffin-embedded sections were cut to 5 μm, and stained with hematoxylin and eosin at Histology Consulting Services Inc. (Everson, WA, USA).

### Polymerase chain reaction (PCR) and sequencing

DNA was extracted (Alaska Dept. of Fish and Game, USA) from the tissue samples using QIAamp^®^ DNA Mini Kit (QIAGEN, Hilden, Germany), with an average output of 94 µg/mL (29–253 µg/mL). Four different PCR protocols were performed (UiT-Arctic University of Norway) as specified in Table 2. Extracted DNA from tissue from a sheep with CE and verified ORFV infection was used as positive control. Deionized and filtered water was used as a negative control.

**Table 2:** PCRs targeting four different gene regions were used to amplify parapoxvirus-specific DNA from tissue samples obtained from 16 clinical cases of contagious ecthyma in five species of Alaskan ruminants (2001–2012)

Gene	Acronym	Primers	Product (bp)	References
Putative viral envelope antigen	*B2L*	PPP-1 → 5′-gtc gtc cac gag cag ct-3′ PPP-4 → 5′-tac gtg gga agc gcc tcg ct-3′	594	[23–25]
Granulocyte–macrophage-colony-stimulating factor (GM-CSF)/interleukin-2 inhibition factor	*GIF*	GIF 5 → 5′-gct cta gga aag atg gcg tg-3′ GIF 6 → 5′-gta ctc ctg gct gaa gag cg-3′	408	[26, 27]
Viral interleukin 10 orthologue	*vIL*-*10*	vIL-10-3 → 5′-atg cta ctc aca cag tcg ctc c-3′ vIL-10-4 → 5′-tat gtc gaa ctc gct cat ggc c-3′	300	[25, 28, 29]
A-type inclusion	*ATI*	ATIF → 5′-agc cac agc act ttc gta cat-3′ ATIR → 5′-cgc gtt tgt gtt cgt tgg at-3′)	604	

The PCRs were run in a DNA Thermal Cycler PE9700 (PerkinElmer Norge AS, Kristiansand, Norway). The PPP 1 and PPP 4 primers targeting the *B2L* gene [23, 25], assumed to be able to detect all parapoxviruses, are based on the *B2L* gene sequence of the ORFV strain NZ2, which encode a homologue of the vaccinia virus major envelope antigen p37K gene [24]. The *GIF* gene encodes a protein that inhibits the granulocyte–macrophage-colony-stimulating factor (GM-CSF) and interleukin-2 and is found only in parapoxviruses, where it represents an important virulence factor [26]. Amplification of parts of the *GIF* gene may thus be useful for detection and virus species differentiation of parapoxviruses, since variation in this gene exists between different parapoxviruses [25, 27]. The viral interleukin 10 orthologue (*vIL*-*10*) [28] needs to have a close similarity to the interleukin-10 of the host for effective virus replication. PCR targeting this gene may be useful for genus affiliation, and nucleotide sequencing of the PCR amplicon suitable for virus characterization [25]. A-type inclusion bodies (ATIs) are large, well-defined proteinaceous bodies that develop late in the viral replication cycle in cells infected with certain poxviruses. The gene encoding the ATI protein homologue in parapoxviruses has been identified at position 107356–108252 of the complete ORFV genome. To identify suitable primers we analyzed six full genome sequences of ORFV published in GenBank (AY386263.1, AY386264.1, HM133903.1, DQ184476.1, KP010354.1, KF234407.1). Alignment was done by LAGAN software [30] and Primer-BLAST [31] to identify specific primers. The reaction mixture and cycling profile was conducted as described for the *B2L* PCR [25], with a Tm of 60 °C.

PCR products were separated by electrophoresis in a 1.2% agarose gel containing 0.005% ethidium bromide, with a separation time of 1.5 h at 6.5 V/cm. Unused dNTP and primers were enzymatically removed from the generated PCR products (ExoSAP-IT™; Amersham Pharmacia Biotech, Sweden), followed by the sequencing protocol for the BigDye^®^ Terminator v3.1 Cycle Sequencing Kit (Applied Biosystems, Norway) [25]. The sequence was determined with the Applied Biosystems 3130xl Genetic Analyzers (Applied Biosystems, Warrington, UK).

### Phylogenetic analysis

The evolutionary history for the *B2L*, *GIF*, *vIL*-*10* and *ATI* genes was inferred by using the Maximum Likelihood method based on the Tamura 3-parameter model and Gamma-distribution [32]. Initial tree(s) for the heuristic search were obtained automatically by applying Neighbor-Join and BioNJ algorithms to a matrix of pairwise distances estimated using the Maximum Composite Likelihood (MCL) approach, and then selecting the topology with superior log likelihood value. The tree with the highest log likelihood is shown. Phylogeny was tested by Bootstrap method with 1000 bootstrap replications. Trees were drawn to scale, with branch lengths measured in the number of substitutions per site. Codon positions included were 1st + 2nd + 3rd + Noncoding. All positions containing gaps and missing data were eliminated, leaving a total of 470 nucleotides for *B2L*, 268 nucleotides for *GIF*, 214 for *vIL*-*10* and 548 for *ATI* in the final dataset. Evolutionary analyses were conducted in MEGA7 [33].

## Results

### Gross pathology and histopathology

**Fig. 2:**
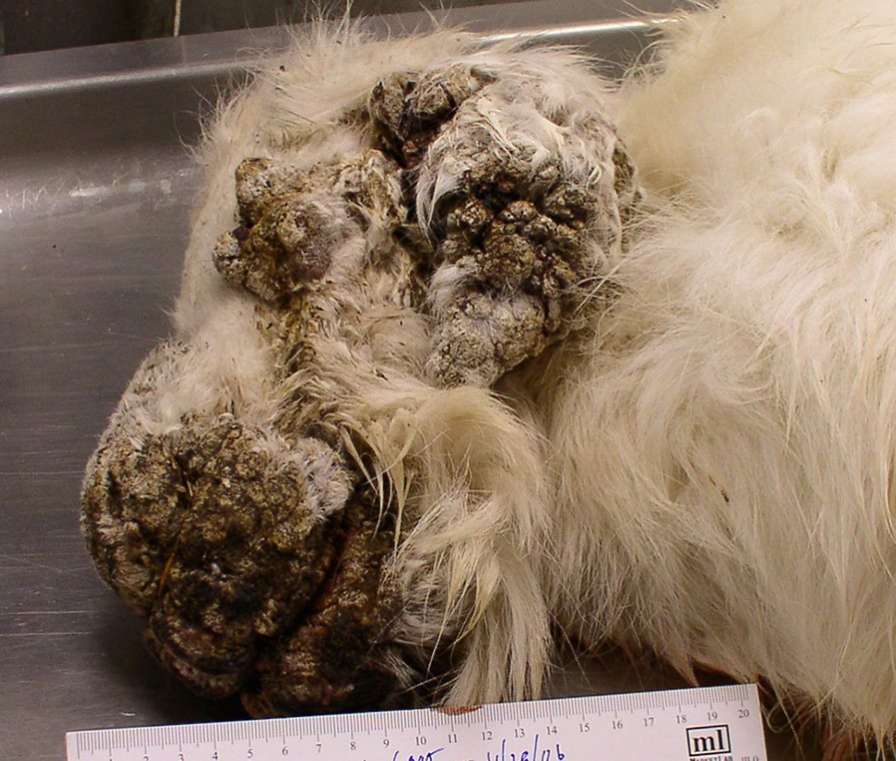
Contagious ecthyma in a mountain goat (*Oreamnos americanus*) having massive proliferative lesions over the muzzle, eyes and ears: case no. 6

**Fig. 3:**
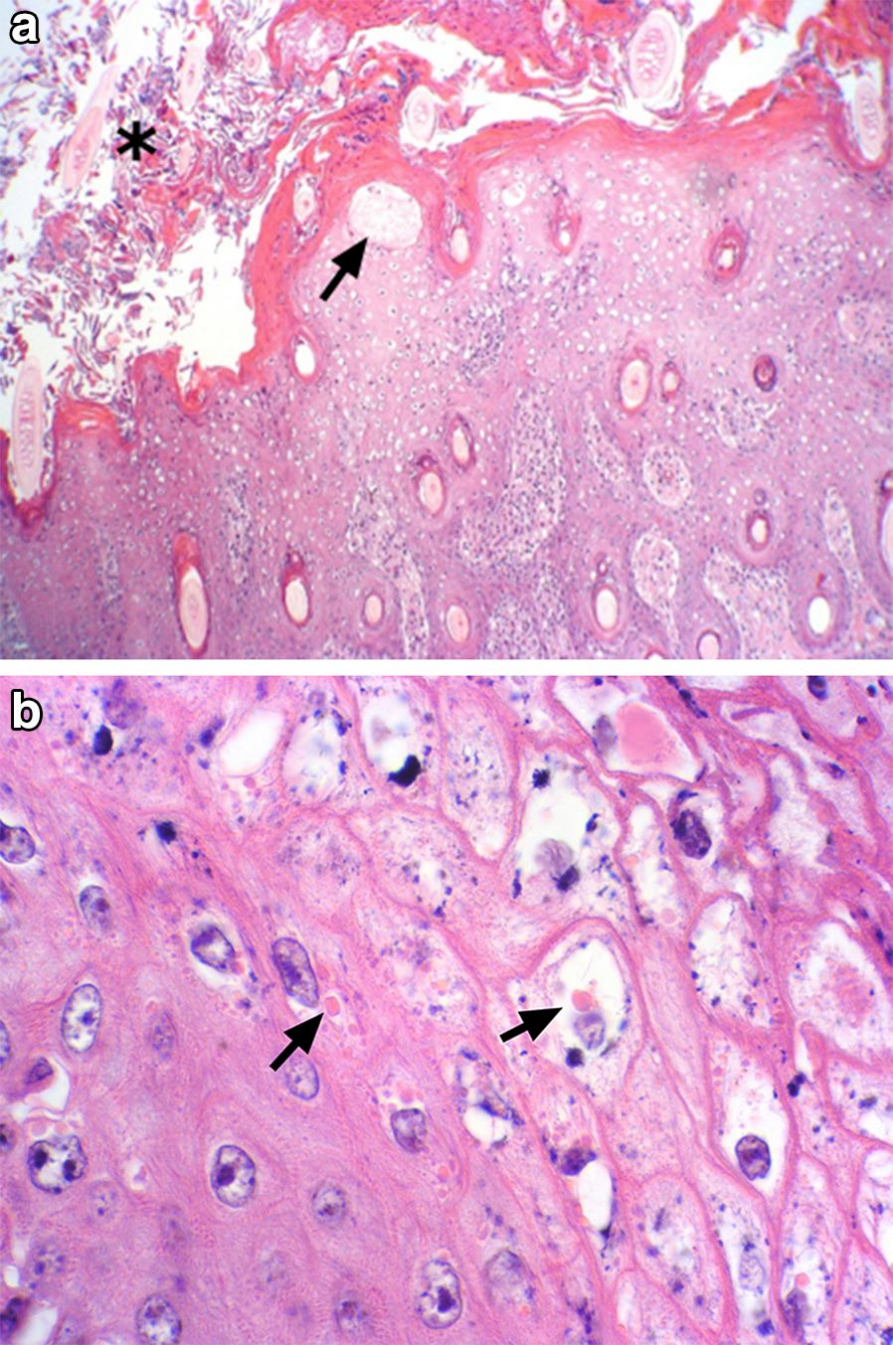
Photomicrograph of contagious ecthyma lesions in a mountain goat, case no. 6. **a** Section of the lip with ulcerative and proliferative dermatitis with marked acanthosis, hyperkeratosis, hydropic degeneration of the stratum spinosum, formation of subcorneal vesicles (arrow) and microabscesses below the stratum corneum, and heavy colonization with mixed bacteria (asterisk) (Obj. ×10). **b** Pale intracytoplasmic inclusion bodies (arrow) could be identified in stratum spinosum and granulosum (Obj. ×100)

In mountain goats, most mucocutaneous regions were affected, including nares, lips, palpebrae, anus, prepuce or vulva as well as coronary bands with lesions extending proximally. In some cases, the pinnae were also involved with masses that nearly obliterated the normal anatomy (Fig. 2). The masses were solid and proliferative, covered by dark friable crusts, which on section showed mottled tan and white tissues with pockets and draining tracts of purulent exudate. When perineum was involved, the proliferative masses coalesced and the vaginal and anal orifices were obliterated. Resolving lesions around the palpebrae and lips presented with edematous, pink to red skin, denuded of hair. *Fusobacterium necrophorum* was most often isolated when bacterial cultures were attempted. Histopathology was consistent with those seen in domestic sheep with CE [4], characterized by ulcerative and proliferative dermatitis (Fig. 3a) with marked acanthosis, hyperkeratosis, hydropic degeneration of the stratum spinosum, formation of subcorneal vesicles and microabscesses, keratohyalin granule clumping and rare small, intracytoplasmic eosinophilic inclusion bodies (Fig. 3b). In some tissues, colonies of mixed bacterial species were seen, with massive influxes of neutrophils and development of granulation tissue.

**Fig. 4:**
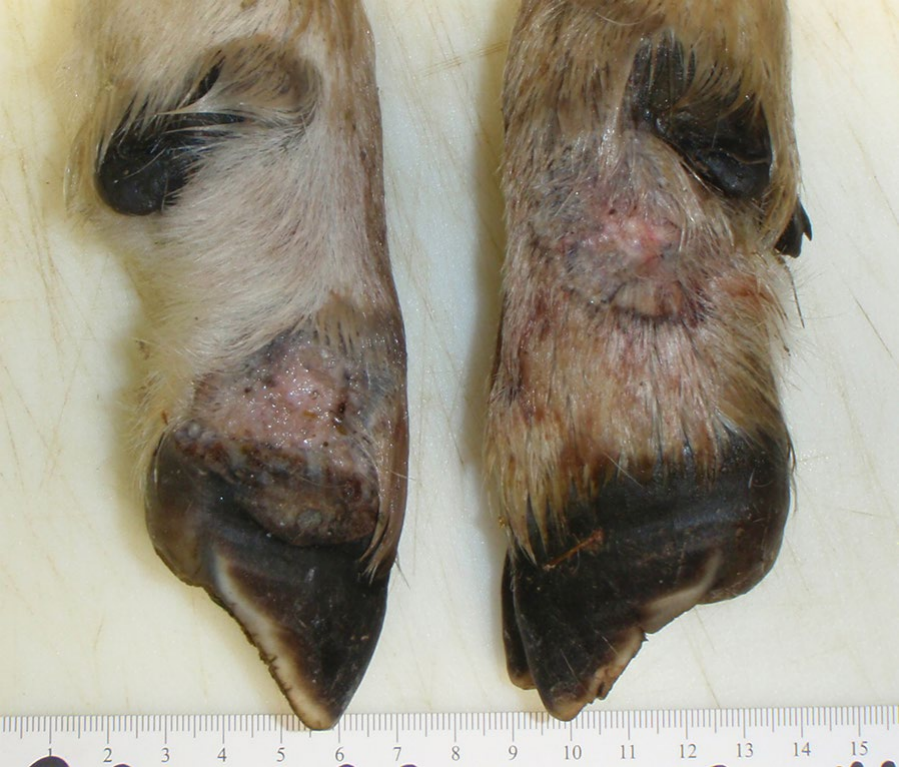
Contagious ecthyma in a young Dall’s sheep (*Ovis dalli dalli*) with mildly proliferative lesions and secondary bacterial infections, mainly restricted to the coronary bands and the skin below the dew claws: case no. 11

For lambs and juveniles of Dall’s sheep, the gross lesions were similar to those described for mountain goats, but not as extensive and the ears were not typically involved. Adult sheep had crusted, proliferative lesions covered by thick crusts around the mouth, similar to what is typical in domestic sheep, as well as, in some cases, at the coronary bands and occasionally in the inguinal region. One young lamb had mildly proliferative lesions oriented around the hooves at the coronary band as well as on the skin below the dew claws (Fig. 4). Histologically, the appearance was typical of parapoxvirus infections as described above for mountain goats, but with faint intracytoplasmic inclusions in only few sections, whereas bacterial colonization of the crusts was common.

For one of the muskox cases, a 5 years old male killed by predators, only the feet and head were available, the latter presenting a ‘roughening’ of the skin of the external nares. For the second muskox case, an adult female, only partial and partly autolyzed remains were available for examination, demonstrating multifocal lesions around the nares consistent with erosions or ulcers. There was a whitish, proliferative mass in the interdigital space, which was the source of the sample included in this study (Table 1, case no. 9). On histopathology there was a prominent layer of vacuolated keratinocytes in the stratum corneum, and multiloculated vesicles and pustules. Much of the surface of the stratum corneum was eroded, roughened and had affixed foreign material, and cross sections of nematode larvae and bacteria. In the submucosa, mild chronic inflammation was present.

**Fig. 5:**
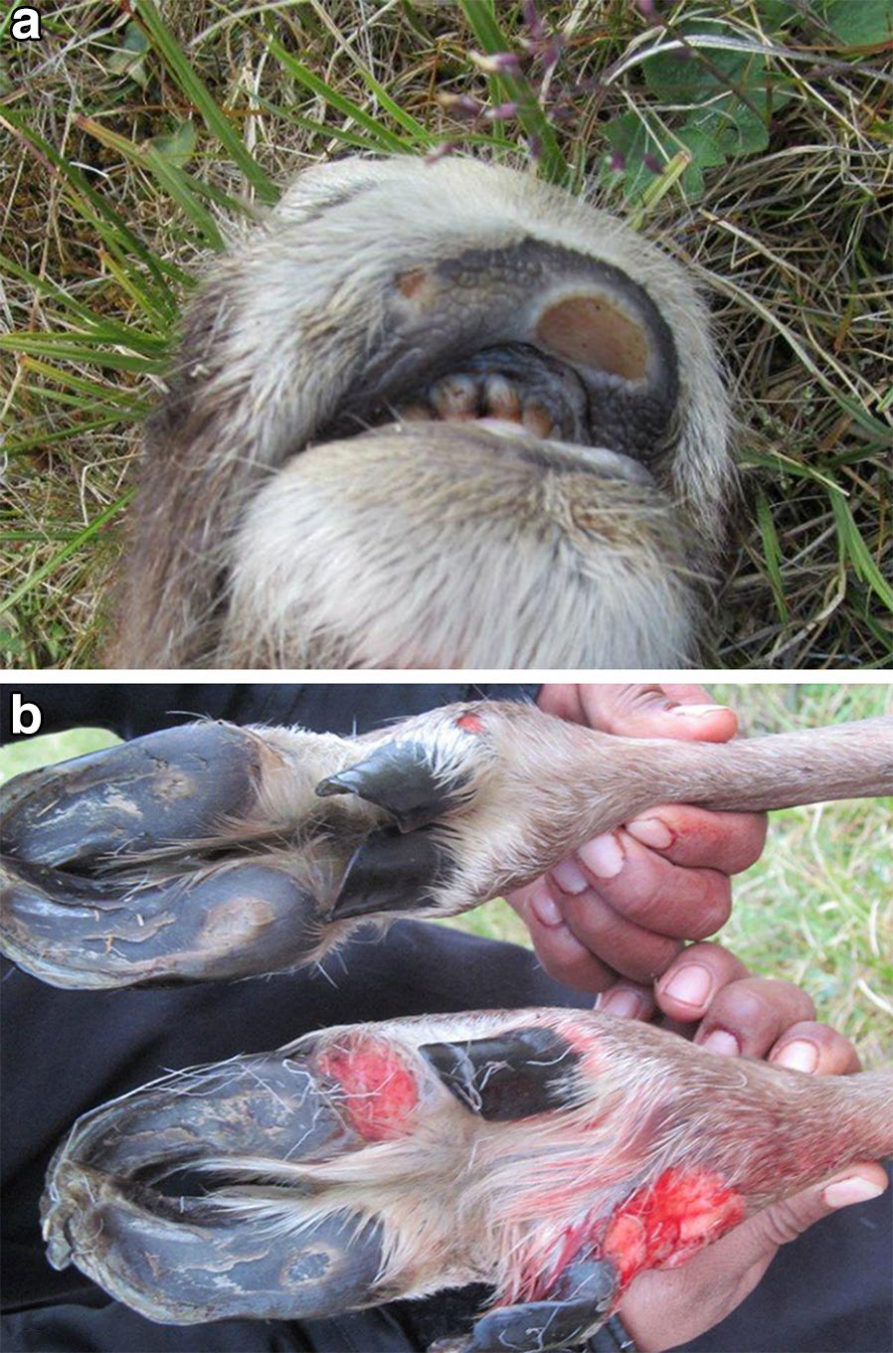
Contagious ecthyma in a caribou (*Rangifer tarandus granti*) with **a** ulcerative lesions in the muco-cutaneous interface of the upper lip and **b** the plantar side of the hooves/coronary bands and carpal joints: case no. 16

From the caribou case, a hunter harvested, yearling, female, only the lower front legs were submitted for examination. However, the hunter reported an animal in poor body condition, with lymphadenopathy and one large (25 × 20 mm) and one smaller (5 × 6 mm) ulcerative lesion on the upper lip (Fig. 5a). On the left leg there was a 20 × 5 mm ulcer behind the heel of the lateral claw and another 15 × 9 mm ulcer above the dew claw (Fig. 5b). The leg demonstrated decreased bone and muscle mass and had overgrown dew claws consistent with disuse atrophy. On the right leg, there was a large ulcer (30 × 30 mm) near the heel, and an extensive deep ulcer (58 × 54 mm) on the lateral claw. The foot was enlarged at phalanx 1 and 2 due to extensive fibrous proliferation of the subcutaneous tissue. There was purulent exudate in the deep subcutaneous tissues and tendon sheaths from which *Trueperella pyogenes* was isolated by culture. Histopathology, conducted on previously frozen tissue, revealed changes typical of a chronic ulcer with focal areas denuded of epidermis, bordered by a broad zone of epidermal hyperplasia and hyperkeratosis as well as surrounding and underlying inflamed granulation tissue. Epithelium adjacent to the ulcers were colonized by coccobacilli and segmented fungal hyphae. The deep dermis had multifocal, perivascular to diffuse infiltration of lymphocytes and plasma cells. In the areas of tendon and tendon sheaths, there was loss of synovium and accumulation of fibrin, neutrophils, macrophages and mixed bacteria with chronic active inflammation extending into surrounding connective tissue.

The Sitka black-tailed deer tissue was obtained from a hunter-harvested adult female from Kodiak Island. The hunter submitted a large hairless spherical mass 14 × 14.2 × 9.5 cm weighing 959 g that was covered with dark skin and had been located just to the right of the base of the tail. A similar subcutaneous 9 × 7.5 × 6 cm mass weighing 176 g was present left of the base of the tail. On cut section, the tissue was firm, white, and fibrous. Histology revealed that the mass was covered by an epithelium with mild acanthosis, hyperkeratosis and cystic hair follicles (Additional file 1a), and was composed of interweaving bundles of collagen with a minimum number of nuclei, consistent with a fibroma (Additional file 1b). The center of the mass was necrotic and mineralized.

### Polymerase chain reaction (PCR)

All the four PCRs, targeting the genes *B2L*, *GIF*, *vIL10* and *ATI*, were able to generate amplicons of expected size from tissue samples from all the three samples from each case and from all the 16 cases, as well as from the positive control, visualized by gel electrophoresis (data not shown). The negative control remained negative throughout the study.

### Phylogenetic analysis

**Fig. 6:**
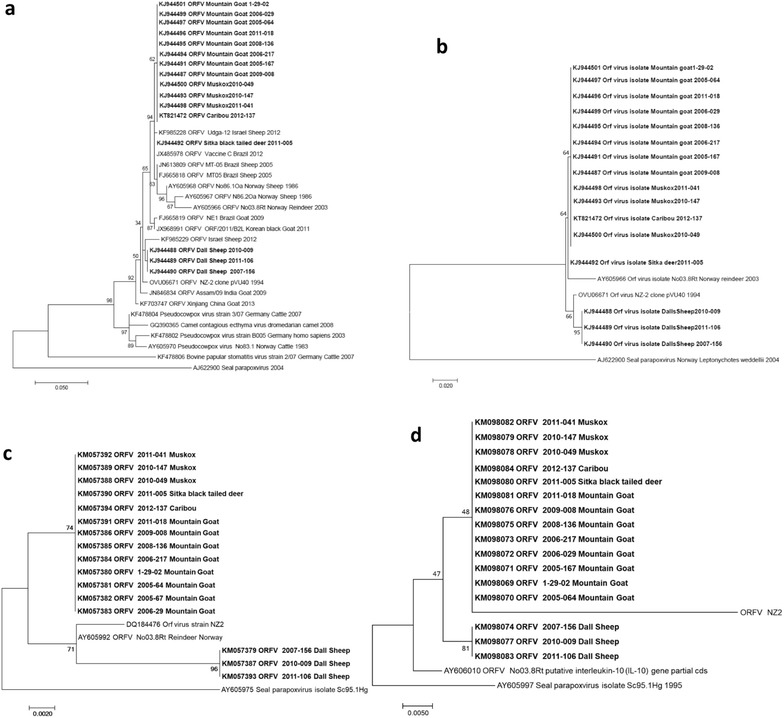
Phylogenetic comparison of the parapoxvirus sequences generated from tissues of mountain goat (*Oreamnos americanus*), Dall’s sheep (*Ovis dalli dalli*), muskox (*Ovibos moschatus*), caribou (*Rangifer tarandus granti*) and Sitka black-tailed deer (*Odocoileus hemionus sitkensis*) from Alaska (this study) with other parapoxvirus sequences (GenBank), including the type strain of ORFV (NZ-2). **a** Comparison of the 470 nucleotides from the putative viral envelope gene *B2L* compared to similar sequences from GenBank, and **b** with each other, including a reindeer (*R. t. tarandus*) isolate and a reference strain. **c** Comparison of the sequences obtained from the GM-CFS IL 2 gene (*GIF*). **d** Comparison of the sequences obtained from the putative interleukin 10 gene (*vIL10*) (see Table 1 for reference to sequence identity)

The trees with the highest log likelihood are presented. The percentage of replicate trees in which the associated taxa clustered together in the bootstrap test (1000 replicates) are shown next to the branches. The phylogenetic analysis of the PCR amplicons from the *B2L* gene region (Fig. 6a) of the 16 cases, revealed that they all were ORFV, grouping them among other ORFV isolates, and distinguishing them from other parapoxvirus species. Comparing these 16 sequences with each other (Fig. 6b) revealed that the Dall’s sheep formed a separate cluster, having the reference strain of ORFV (NZ2) as its closest neighbor, whereas sequences obtained from the other species were identical except for the sequences from the black-tailed deer, which interestingly was closer to a sequence from reindeer (*R. t. tarandus*, Norway) than the caribou and the other species represented in this study. The *GIF* and *vIL*-*10* gene sequences (Fig. 6c, d) revealed that the Dall’s sheep again formed a separate cluster, whereas the other cases and species included in the study had identical sequences compared to each other, for both genes.

The phylogenetic analysis of the sequences generated from the ATI gene region showed no diversity between the cases included in this study, i.e. all derived sequences were identical, with the exception of a positive control derived from an ORFV infected sheep from Norway (data not shown).

## Discussion

This is the first report on CE among free ranging ruminants in Alaska with support from virus characterization by sequencing and phylogeny, and the first documentation of this disease in caribou and Sitka black-tailed deer.

The diagnosis of CE is often based on clinical and pathological observations. For the cases included in this study, these findings generally resembled the ones that have been described for CE in sheep and goats [4]. However, the findings in mountain goats were particularly severe, characterized by proliferative and well vascularized lesions, which in two cases were fatal, due to trauma and severe loss of blood. The lesions seen in Dall’s sheep were typical and consistent with previous reports for this species [9] and for domestic sheep, being more severe in lambs as compared to adults. The lesions in the muskoxen were subtle and not the cause of mortality, which is in contrast to a disease outbreak reported in free-ranging muskoxen in Norway, where 18 calves and one yearling died or were euthanized due to severe CE [10]. The Sitka black-tailed deer was unusual with just two large spherical lesions near the tail that were histologically a fibroma, and the typical parapoxvirus-associated lesions were not found at histology. Since it occurred in Kodiak, a geographical region with a mountain goat population that had been trans-located from southeast Alaska, a transmission from mountain goats seemed to be the most likely origin of the virus in that case. However, the PCR sequences generated for the *B2L* gene indicated a difference between the Sitka black-tailed deer as compared to the mountain goat sequences. The caribou had lesions similar to those that have been reported from semi-domesticated Eurasian tundra reindeer [7], but was also severely debilitated from a concomitant tendosynovitis. Although the latter condition appeared to be caused by a secondary bacterial infection, ORFV was detected also in these structures by PCR. The caribou occurred sympatric with muskoxen, and the source of infection might have been contact with infected muskoxen. There are no indication that the clinical cases were linked by transmission or a disease outbreak since the cases were well separated over space and time. There were six cases in mountain goats from the same game management unit (GMU) (1C) but these were diagnosed over 5 different years (Table 1).

Comparing the PCR amplicons generated by four different PCRs from tissue samples from five different animal species, separated geographically over most of the Alaskan land area, and sampled over a time period of more than a decade (2001–2012) revealed a high degree of homology. In fact, only ORFV sequences from the Dall’s sheep formed a separate cluster, whereas ORFV sequences generated from the clinical cases in the other ruminant species showed a high degree of homology (Fig. 6), and thus raise the issue of possible contamination. Contamination may occur during necropsy, tissue subsampling or during any of the extraction and PCR steps during the investigation, and may also originate from PCR amplicons in the laboratory from previous investigations. In this case, the necropsies were not conducted simultaneously but during the whole time period and in two different laboratory buildings. We did not include a negative control sample (i.e. a tissue sample known not to contain ORFV) along with the tissue samples from the clinical cases during the extraction step to indicate if inter-sample contamination may have occurred, but the tissue sub-sampling was conducted with aseptic techniques to avoid such cross-contamination. Further, the DNA extractions were conducted in a different institution (University of Alaska, Fairbanks), and the analyses were conducted in separate labs for PCR, post-PCR and sequencing (UiT-Arctic University of Norway), diminishing the risk of contamination of the samples with PCR products. In addition, and since the PCR protocols addressing the *B2L*, *GIF* and *vIL10* genes have been used at UiT-Arctic University of Norway before, suggesting a potential risk for laboratory contamination of PCR amplicons from previously run PCRs, we developed a new set of PCR primers targeting the *ATI* gene, a genomic region that had never been amplified before in that laboratory. In contrast to the results from the three other genes, where Dalls’s sheep formed a separate cluster, the ATI PCR generated identical sequences from all the 16 cases. The fact that the generated sequences from the *B2L*, *GIF* and *vIL10* genes identified the Dall’s sheep as a separate cluster, and that all the sequences were in fact different from the ORFV isolates and samples we have hitherto worked with in the laboratory (Fig. 6, Accession Nos. AY605968, AY605967 and AY605866), advocated for excluding contamination as an explanation for the high homology among the generated sequences.

The phylogenetic analysis of the *B2L* gene region showed that all the 16 clinical cases included in this study had ORFV-specific DNA in affected tissues, and that the samples from Dall’s sheep formed a separate cluster for all the three genes, *B2L*, *GIF* and *vIL10*. This was, however, more obvious for the *B2L* and the *GIF* genes than the *vIL10* gene, since for the latter (Fig. 6d), the branch likelihood at the separation point between the Dall’s sheep and the other cases in this study, was only 47 and thus weak, suggesting that this separation was likely, but not significant for this specific region. For the *GIF* gene sequences (Fig. 6c), it was noted that the long branch for the Dall’s sheep cluster indicated a high amount of base substitutions within this important immune evading gene region. Those findings are in accordance with a previous study where restriction enzyme analysis (*Kpn*I) distinguished between two Alaskan isolates obtained from a Dall’s sheep and a muskox [34]. However, the phylogeny presented here demonstrated that the high degree of homology between the different amplicons generated from ORFV for all the four PCRs restricts their value as epidemiological tools, i.e. to address the source of infection and contacts between animal populations.

The high degree of genetic conservation found among the ORFV sequences in this study may seem surprising but is not unique. In fact, a comparison between the PCR amplicon sequences generated in this study with a variety of similar sequences from ORFV obtained from different animal species, time periods and regions of the world (Fig. 6a), clearly illustrates how conserved these gene regions are. Similar features have been identified also for other poxviruses previously. A comparison between a tanapoxvirus isolated from a human case in the Tana River Valley, Kenya, in 1957 (TPV-Kenya) and an isolate from an infected traveler in the Republic of Congo in 2004 (TPV-RoC) was conducted [35]. Although isolated 50 years apart the two genomes were highly conserved, differing at only 35 of 144,565 nucleotide positions (99.98% identical). With the results generated by our study, it is suggested to use sequences from larger and more variable gene regions to be able to distinguish between most of the ORFV’s circulating in Alaskan wildlife.

In Norway, it has been demonstrated that ORFV has been transferred from domestic sheep to semi-domesticated reindeer (*R. t. tarandus*) [7] and muskoxen [10]. In an experimental trial, it was also shown that domestic sheep were susceptible to oral inoculation of ORFV isolated from reindeer [36]. In Alaska, domestic sheep are present in areas in which contact could have occurred with the wild ruminants. It has been speculated that ORFV has been enzootic in the populations of Dall’s sheep in Alaska for a long time, and that CE was seen in muskoxen from the Nunivak Island already in the 1930s [9]. This study shows that most of the wild ruminant species in Alaska can carry and be affected by ORFV, i.e. developing CE. These findings are thus relevant for management purposes, since the ORFV is transmissible to people as well as to domestic sheep and goats.

CE is an often neglected zoonosis, presumably because sheep and goat farmers, the main groups at risk worldwide, are familiar with the disease and do not often seek medical assistance upon infection. This is also illustrated in a recent review of zoonotic infections in Alaska, in which *Brucella* spp., *Toxoplasma gondii*, *Trichinella spiralis*, *Giardia* spp., *Cryptosporidium* spp., *Echinococcus* spp., rabies virus and *Francisella tularensis* were listed as relevant zoonotic infections, but not parapoxvirus infections [37]. However, in Canada, ORFV is listed among zoonotic infections relevant for First Nations people in the Arctic [21].

People at certain risk for ORFV infections are farmers of sheep, goats and captive wildlife, hunters of large ruminant game species, and game managers, including veterinarians and diagnostic personnel, and it has been shown that immunocompromised persons are at special risk to develop severe disease. To avoid ORFV infection, people should be aware of the clinical signs and lesions in animals, as presented here, and additional caution is warranted when lesions are present to avoid fomite spread of the virus between animals and people. Personal hygiene including wearing gloves, hand washing and proper heat treatment of potentially contaminated meat and other animal products for consumption are important practices to prevent transmission to people.

## Conclusions

This study describes pathological findings associated with CE in large ruminant wildlife species in Alaska, which was further verified by the presence of ORFV. Sequences obtained from Dall’s sheep formed a separate cluster, but PCRs and sequencing in general revealed a high degree of sequence homology between ORFV from all species and individuals examined, suggesting that other and more variable gene regions should be used for epidemiological purposes. Mountain goat, Dall’s sheep, muskox, caribou and Sitka black-tailed deer all represent popular game species in Alaska, of which approximately 20,000 were hunted in 2015. Since they can carry and be affected by ORFV, these findings call for attention to transmission of ORFV from wildlife to livestock, as well as to hunters, subsistence harvesters, and wildlife biologists.

## Additional file

**Additional file 1.** Photomicrographs of a fibroma in a Sitka black-tailed deer (case no. 15). *a*: The fibroma was covered by an acanthotic epithelium. Histology revealed distended, entrapped follicles (arrow), embedded in a mass composed of spindle cells and collagen bundles (Obj. ×4); *b*: The majority of the mass was composed of interweaving bundles of collagen (arrow) typical of a fibroma (Obj. ×10).
