# Supplementary material for: Orf virus infection in Alaskan mountain goats, Dall’s sheep, muskoxen, caribou and Sitka black-tailed deer
Source: Acta Vet Scand. 2018 Feb 21;60:12. doi: 10.1186/s13028-018-0366-8 (PMC5822636; doi:10.1186/s13028-018-0366-8)

Additional file 1. Photomicrographs of a fibroma in a Sitka black-tailed deer (case no. 15).

a: The fibroma was covered by an acanthotic epithelium. Histology revealed distended, entrapped follicles (arrow), embedded in a mass composed of spindle cells and collagen bundles (Obj. x4); *b*: The majority of the mass was composed of interweaving bundles of collagen (arrow) typical of a fibroma (Obj. x10).

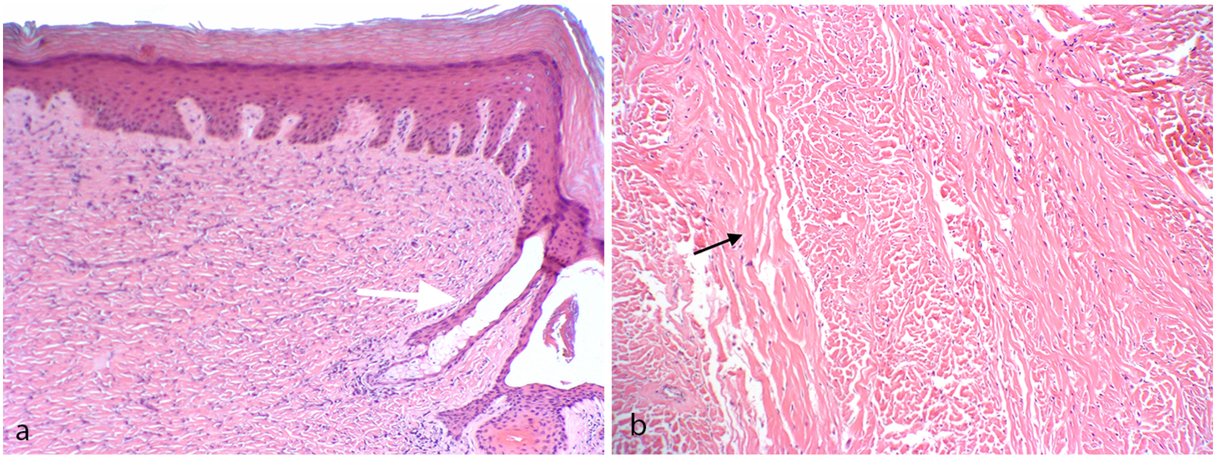

Supplement: Supplementary file 1 — Additional file 1. Photomicrographs of a fibroma in a Sitka black-tailed deer (case no. 15). a: The fibroma was covered by an acanthotic epithelium. Histology revealed distended, entrapped follicles (arrow), embedded in a mass composed of spindle cells and collagen bundles (Obj. ×4); b: The majority of the mass was composed of interweaving bundles of collagen (arrow) typical of a fibroma (Obj. ×10). [file 13028_2018_366_MOESM1_ESM.pdf]
